# Supplementary material for: Optimal non-pharmaceutical intervention policy for Covid-19 epidemic via neuroevolution algorithm
Source: Evol Med Public Health. 2022 Jan 28;10(1):59–70. doi: 10.1093/emph/eoac002 (PMC8841015; doi:10.1093/emph/eoac002)
Supplement: eoac002_Supplementary_Data [file eoac002_supplementary_data.zip › revised_supplementary.pdf]

Supplementary materials for : Optimal non-pharmaceutical intervention policy for Covid-19 epidemic via neuroevolution algorithm

Arash Saeidpour[1,2] and Pejman Rohani[1,2,3]

[1]Odum School of Ecology, University of Georgia, Athens, Georgia, United States of America
[2]Center for the Ecology of Infectious Diseases, University of Georgia, Athens, Georgia, United States of America
[3]Department of Infectious Diseases, College of Veterinary Medicine, University of Georgia, Athens, Georgia, United States of America

Comparison of PMP and Neuroevolution optimal policies

We have derived the optimal control solution via Pontryagin's maximum principle (PMP) and compared the results with neuroevolution optimal policy in Figure S1.

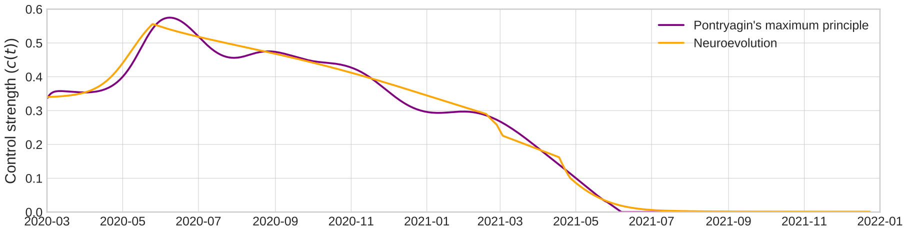

Figure S1: **PMP vs. Neuroevolution intervention policy**

SEIRH model fit to the fatality data

Here we present the SEIRH model fitted on the daily fatality data via particle filtering. The model parameters are described in Table 1 in the main text and the model was fitted to estimate the control strength $c(t)$. We used the fitted model to estimate the initial conditions at different stages of the epidemic for optimal control analysis.

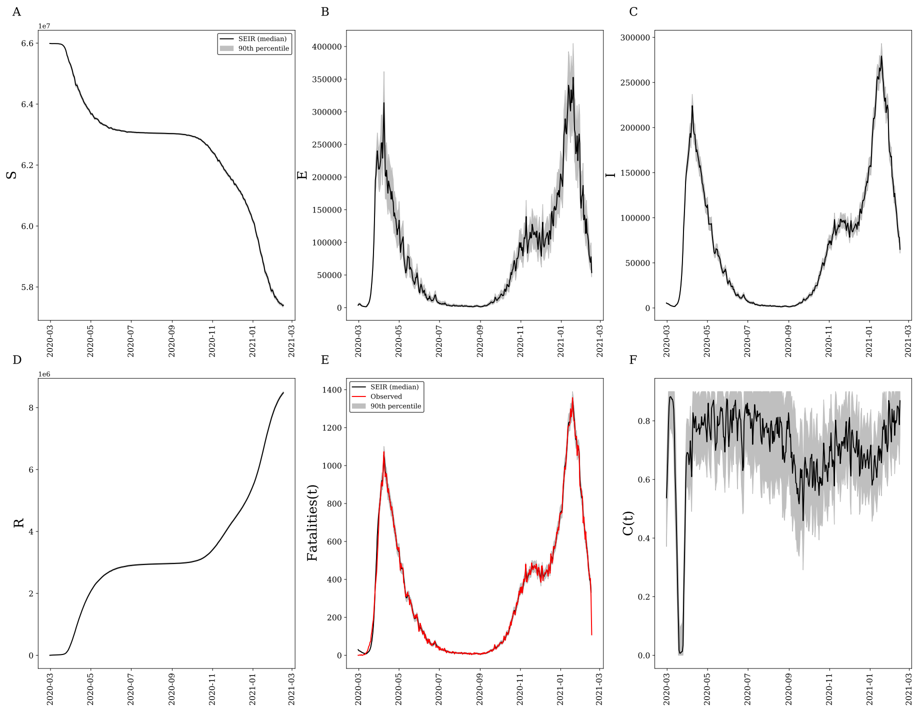

Figure S2: **SEIRH model fitted to the daily fatality data** The figure shows the number of (A) susceptible (B) exposed (C) infectious (D) recovered classes from the fitted SEIRH model. The number of daily fatalities from the data and the model is shown in panel (E). Panel (F) depicts the estimated control strength ($c(t)$). In each panel the black line corresponds to the median of filtering distribution and the shaded area depicts the 90th percentile of filtered particles. The red line in panel (E) presents the fatality data.

Sensitivity analysis

We carried out a sensitivity analysis to investigate the impact of relative weighting of each term in the reward function on the observed optimal policy outcome. This section presents the corresponding results.

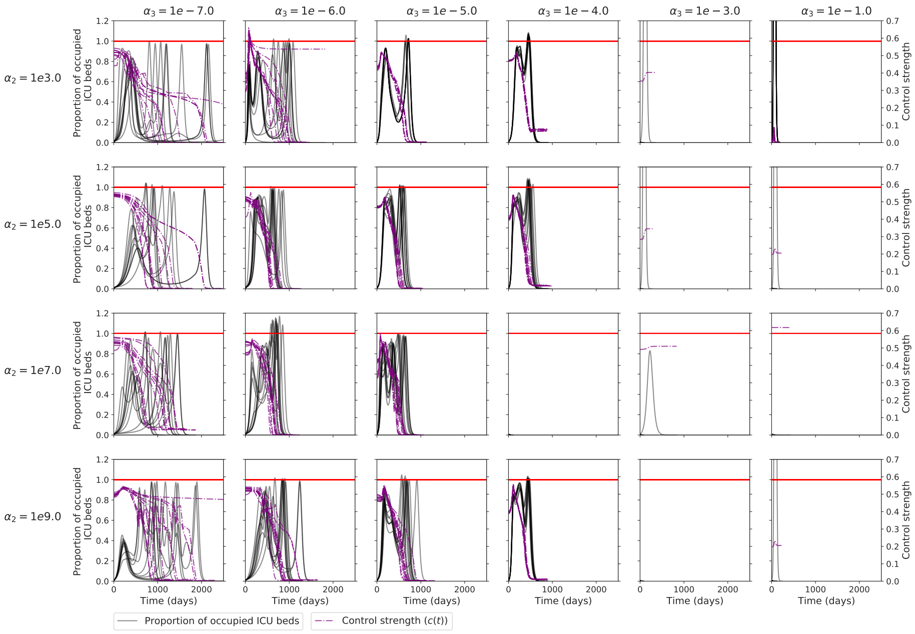

Figure S3: **Sensitivity analysis of reward function parameters** The figure depicts optimal control policy and ICU occupancy trajectory of the 5 most elite agents for each $\{\alpha_2, \alpha_3\}$ combination.

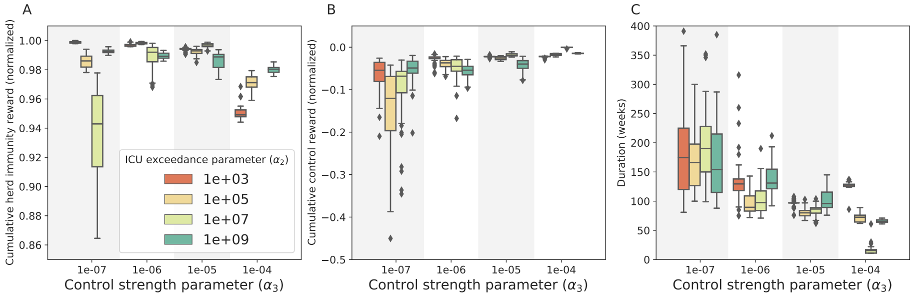

Figure S4: **The optimal control policy is mainly governed by weighting of control strength in the reward function.** The top 50 policy functions for each $\{\alpha_2, \alpha_3\}$ combination is selected and used to reconstruct the epidemic trajectory. Panels denote the aggregated (A) Normalized cumulative herd immunity reward (B) Normalized cumulative control reward (C) Duration of imposing control measures for corresponding $\{\alpha_2, \alpha_3\}$ values.
