# Supplementary figures and images for: Optimal non-pharmaceutical intervention policy for Covid-19 epidemic via neuroevolution algorithm
Source: Evol Med Public Health. 2022 Jan 28;10(1):59–70. doi: 10.1093/emph/eoac002 (PMC8841015; doi:10.1093/emph/eoac002)

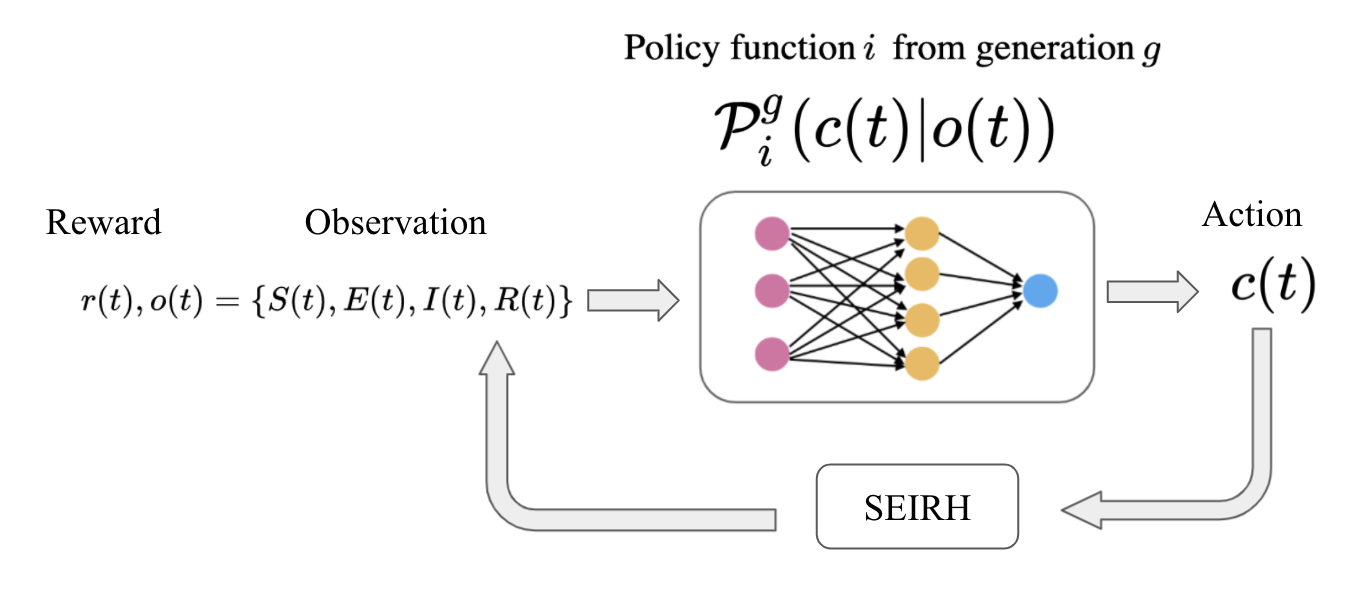

Supplement: eoac002_Supplementary_Data [file eoac002_supplementary_data.zip › Fig2_evo_schematic.png]

A

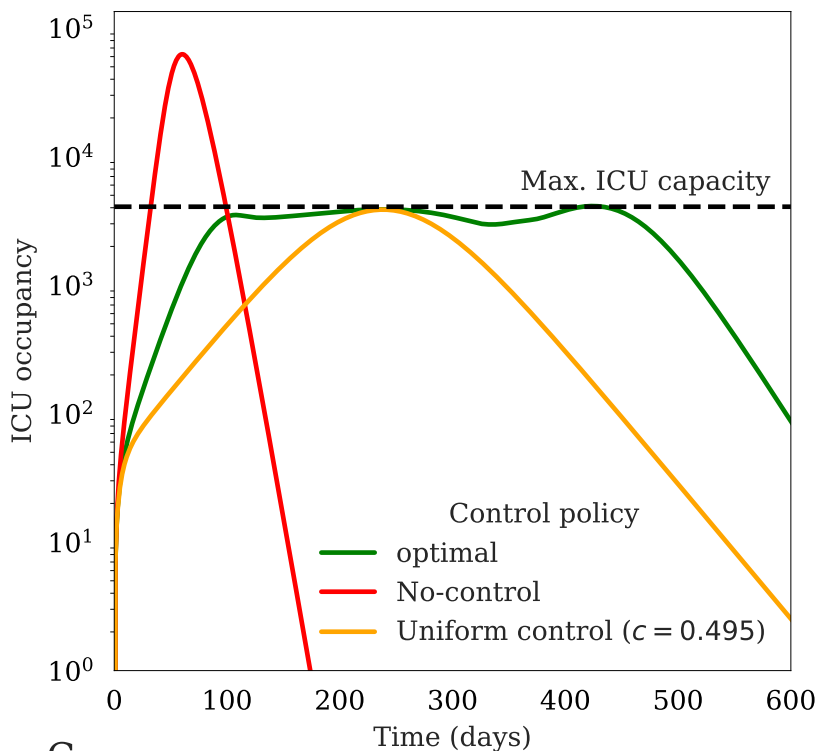

B

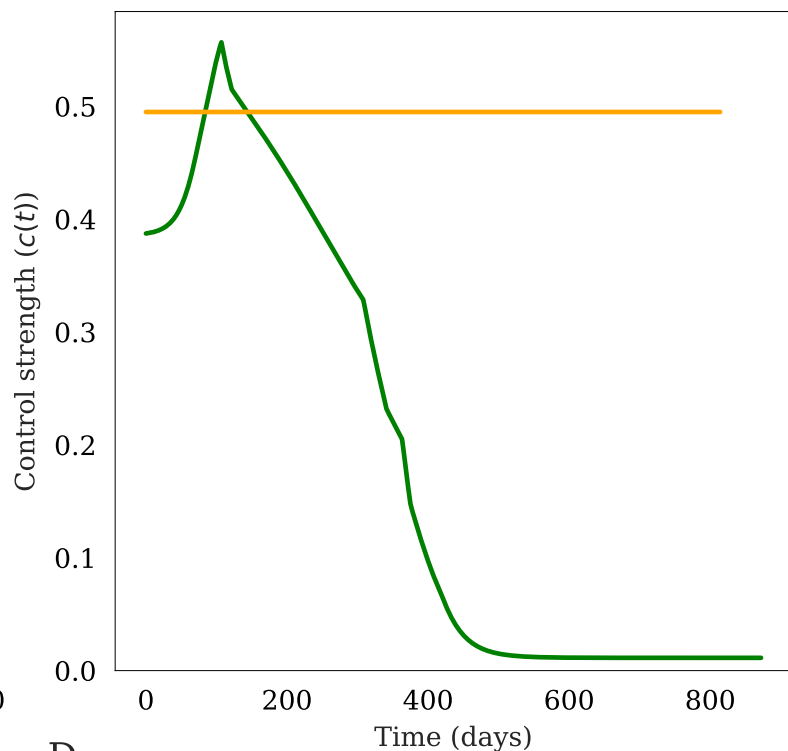

C

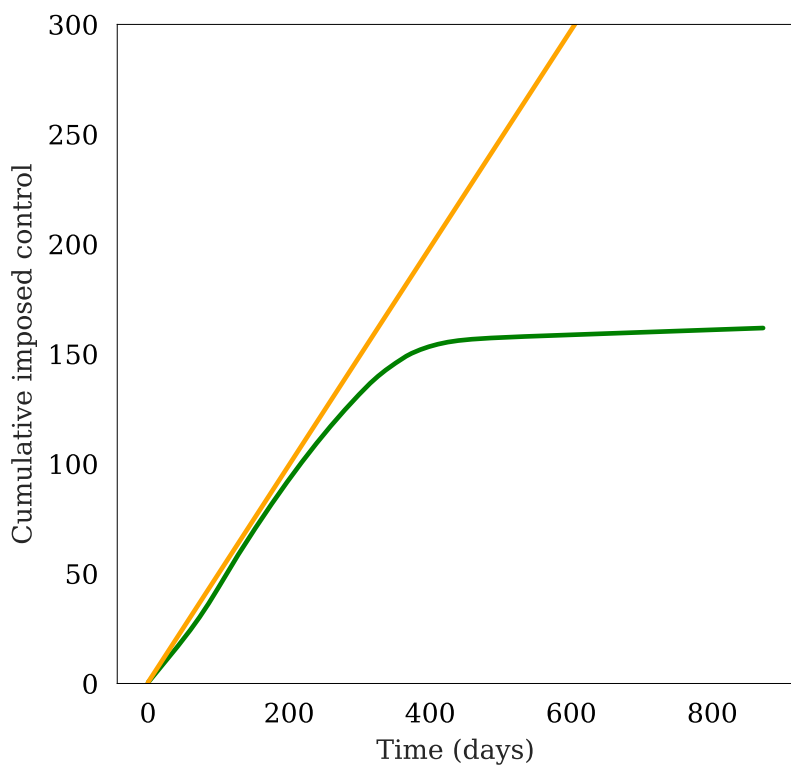

D

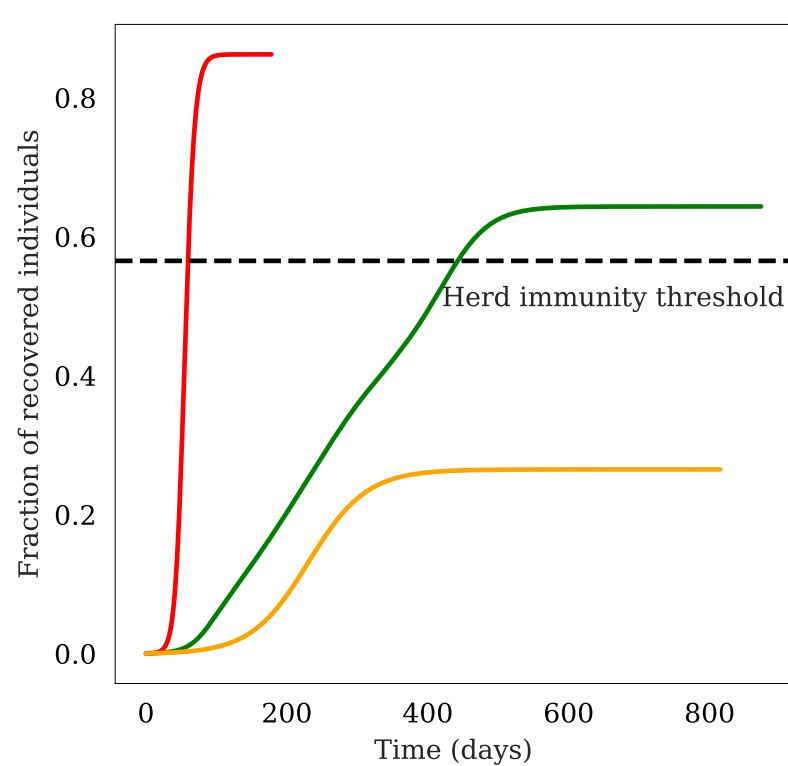

Supplement: eoac002_Supplementary_Data [file eoac002_supplementary_data.zip › Fig3_no_control_vs_uniform_vs_optimal.pdf]

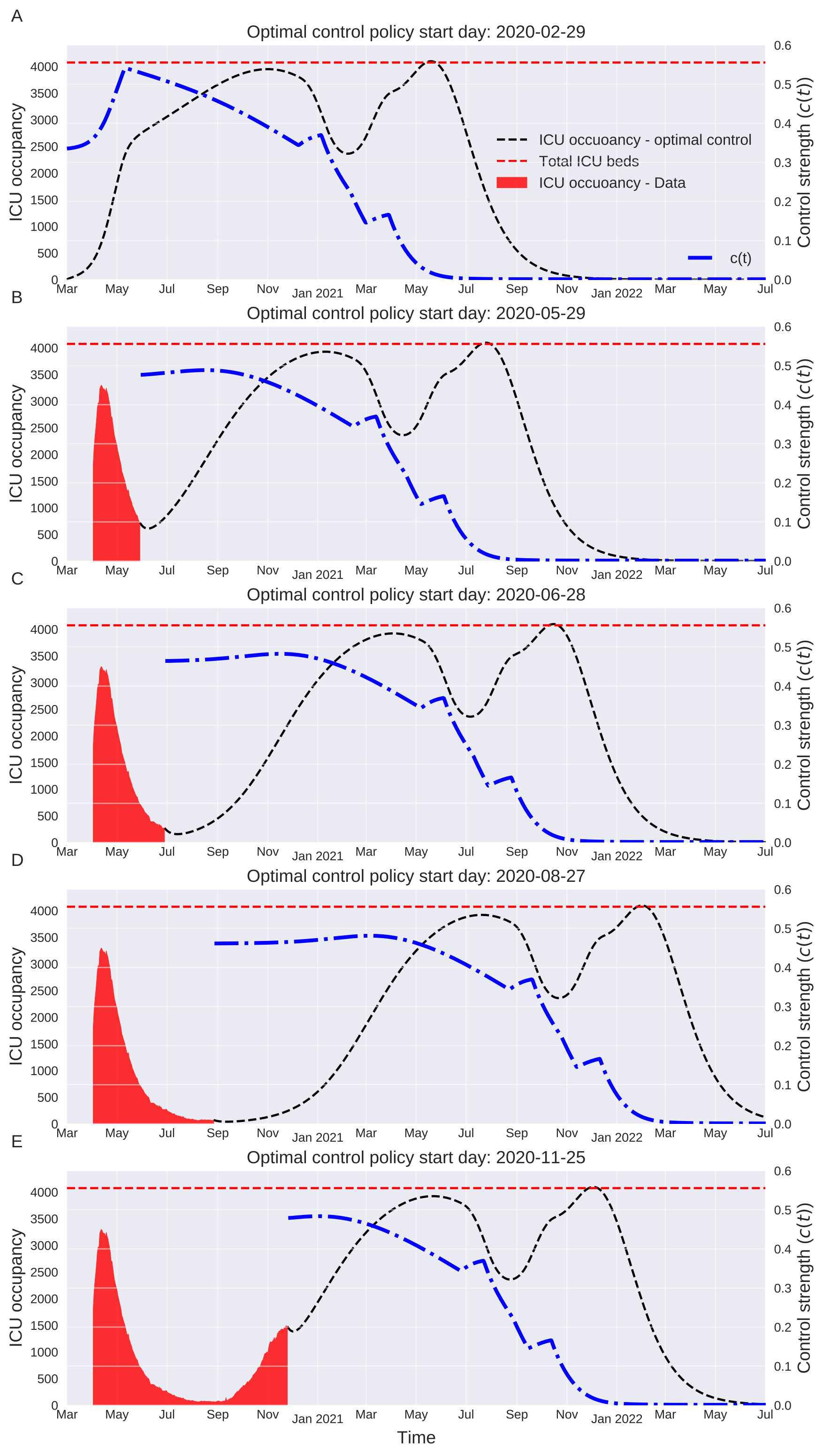

Supplement: eoac002_Supplementary_Data [file eoac002_supplementary_data.zip › Fig4_control_scenarios_1240.pdf]

A

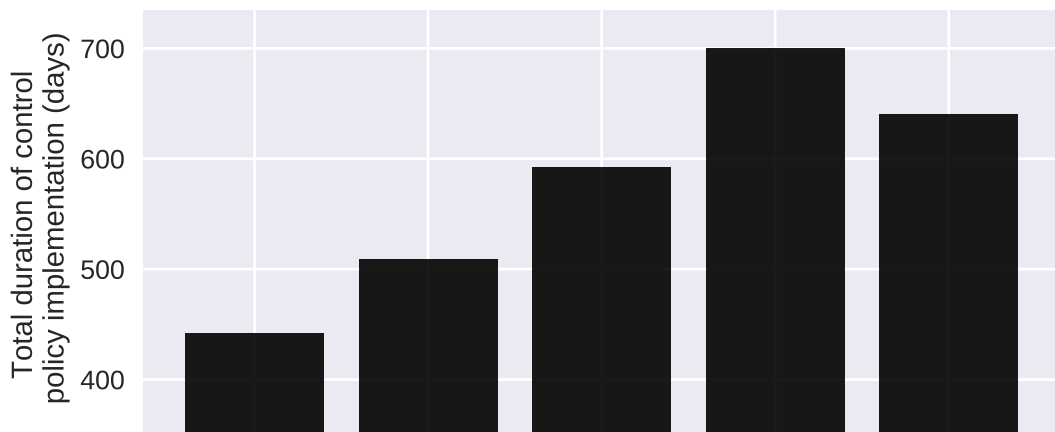

B

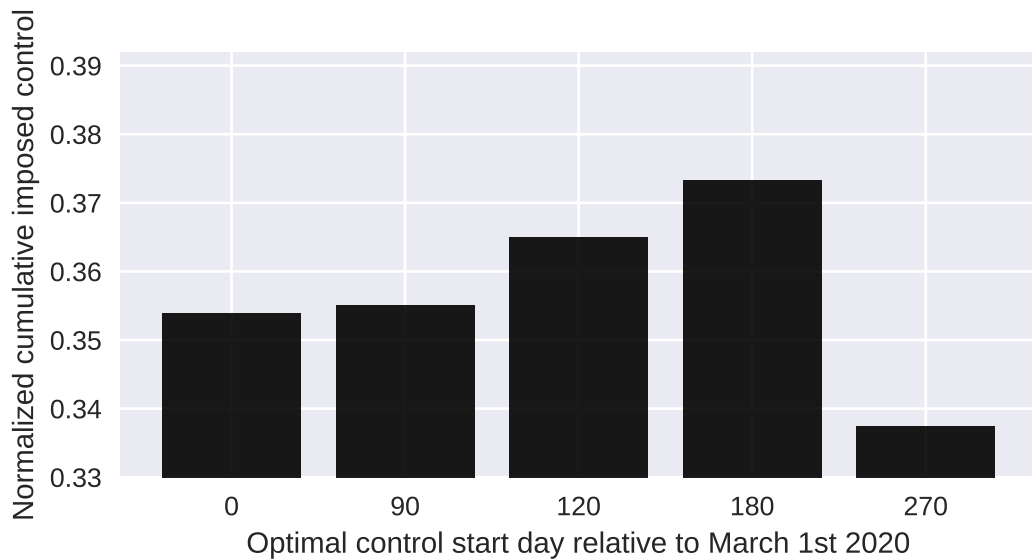

Supplement: eoac002_Supplementary_Data [file eoac002_supplementary_data.zip › Fig5_normalized_cumulative_imposed_control.pdf]

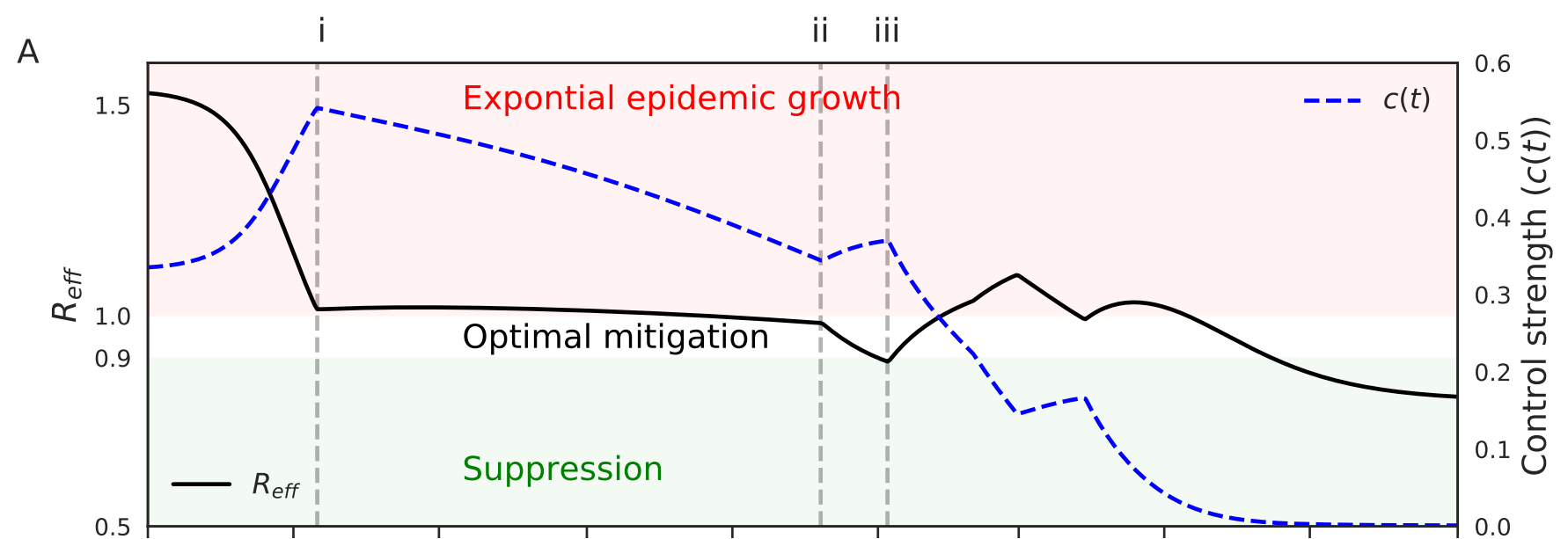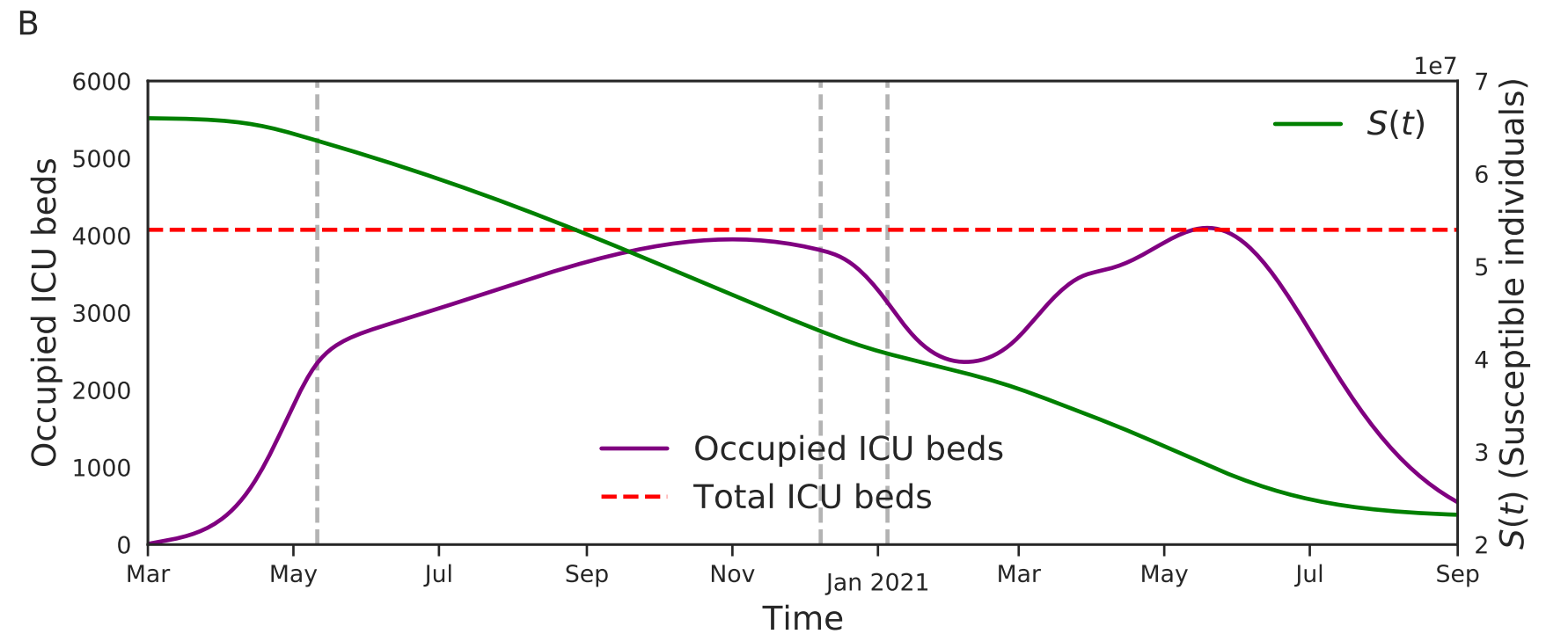

Supplement: eoac002_Supplementary_Data [file eoac002_supplementary_data.zip › Fig6_Reffective_vs_control_policy.pdf]

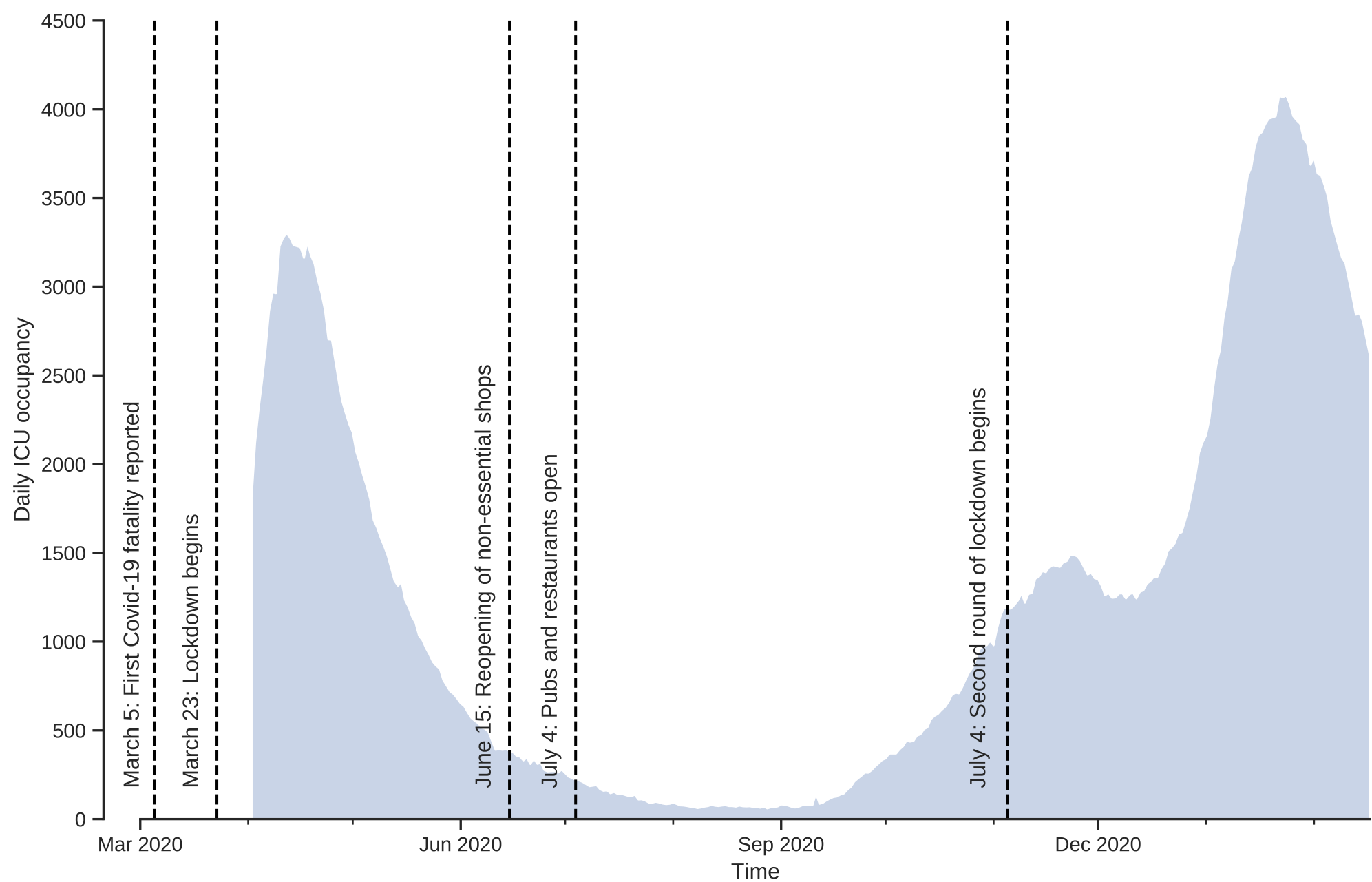

Supplement: eoac002_Supplementary_Data [file eoac002_supplementary_data.zip › Fig1_UK_Covid_timeline.pdf]
